# *Dirofilaria repens* microfilaremia in humans: Case description and literature review

**DOI:** 10.1016/j.onehlt.2021.100306

**Published:** 2021-08-12

**Authors:** Ana Pupić-Bakrač, Jure Pupić-Bakrač, Ana Beck, Daria Jurković, Adam Polkinghorne, Relja Beck

**Affiliations:** a Department of Ophthalmology, General Hospital Zadar, Bože Peričića 5, 23 000, Zadar, Croatia; b Department of Otorhinolaryngology and Maxillofacial Surgery, General Hospital Zadar, Bože Peričića 5, 23 000, Zadar, Croatia; c Veterinary Pathology, 10000 Zagreb, Croatia; d Department for Bacteriology and Parasitology, Croatian Veterinary Institute, Savska cesta 143, 10 000 Zagreb, Croatia; e Department of Microbiology and Infectious Diseases, New South Wales Health Pathology, Nepean Blue Mountains Pathology Service, PO Box 63, Penrith, New South Wales 2751, Australia

**Keywords:** *Dirofilaria repens*, Microfilaremia, Human, Host

## Abstract

**Introduction:**

*Dirofilaria repens* is a vector-borne filaroid helminth of carnivorous animals, primarily domesticated dogs. Humans are considered to be accidental hosts in which *D. repens* rarely reach sexual maturity but induce local inflammation, mainly in subcutaneous and ocular tissues.

**Methods:**

In the current study, we present the detection of multiple adults of *D. repens*, endosymbiont *Wolbachia* sp. and microfilariae by molecular analysis in peripheral tissues and bloodstream of a human host. A subsequent meta-analysis of published literature identified 21 cases of human infection with adult *D. repens* producing microfilariae.

**Results:**

Within the study population, there were 13 (59.09%) males, eight (36.36%) females and, in one (4.55%) case, sex was not reported. A total of 11 (50.00%) cases had subcutaneous dirofilariasis, six (27.27%) had ocular dirofiliariasis, with single cases (4.55% each) of genital, mammary, lymphatic and a combination of subcutaneous and pulmonary dirofilariasis described. In one (4.55%) case, the primary anatomical site of adult *D. repens* could not be found. *D. repens* microfilariae were detected in the local tissue (local microfilariasis) in 11 (50.00%) cases and the peripheral blood (microfilaremia) in 11 (50.50%) cases. Final identification of *D. repens* microfilariae was based on morphological detection in 14 (63.64%) cases, and molecular detection in eight (36.36%) cases.

**Conclusion:**

The results of this study suggest that humans may act as a final host for *D. repens,* however its role as a source of *D. repens* infection is less clear.

## Highlights

• Humans may act as a final host for *D. repens*
• Immunodeficiency is not a risk factor for human *D. repens* microfilaremia.
• Surgical extirpation of adult *D. repens* is a mainstay of treatment.

## 1 Introduction

*Dirofilaria repens* is a vector-borne filaroid helminth of canids with dogs representing the major reservoirs of infestation. The full life-cycle of *D. repens* comprises five larval stages with a prepatent period of approximately 6–9 months [1]. The development of the parasite depends on the availability of competent mosquito species, suitable hosts, adult male and female *D. repens* helminths and the presence of the bacterial endosymbiont, *Wolbachia* sp. [2], the latter required for successful molting and embryogenesis of filariae. Humans acquire *D. repens* infestation in the same manner as dogs after the bite of a mosquito species from the *Culicidae* family [3]. In most cases, however, infective larvae are detected by the body's immune system, leading to destruction of the parasite prior to the infestation being recognised [4]. In some cases, a single larva can survive and molt into a preadult and adult worm. *D. repens* infestation is manifested with local inflammation, mainly in subcutaneous and ocular tissues. Symptoms are usually mild and resolve shortly after surgical extraction of the worm [5]. The development of *D. repens* into a sexually mature worm in humans appears to be uncommon, with antigen sets from both *D. repens* and their endosymbiont *Wolbachia* sp. stimulating specific immunologic reactions that block complete development of the helminth [1]. For this reason, humans were considered to be dead-end hosts for these helminths [6]. In rare cases, *D. repens* can avoid the host's defence mechanisms and reach sexual maturity [7].

In the literature, there are currently 10 case reports of human *D. repens* microfilaremia, and only a few are confirmed with molecular analysis [8]. In the currently study, we report on the detection of multiple adults of *D. repens*, *Wolbachia* sp. and microfilariae in peripheral blood in a human case of *D. repens* filariasis. Additional meta-analysis of the available literature was also conducted identifying additional evidence to support humans as a definitive host for this helminth.

## 2 Case description

Full details of the patient's clinical history are provided in Supplmentary Material 1. Briefly, a 17-year old adolescent athlete presented at an emergency room on 10th December 2019 due to an acute onset of burning pain in the left inguinal region, followed by formation of a shallow subcutaneous nodule measuring 5 × 3 cm in size. The patient was subfebrile (37.2 °C), in a good general condition without any previous relevant medical history. He denied allergies to food and drugs with a skin prick test confirming negative results. Blood tests showed elevation of eosinophil to 16%, but all other parameters were unremarkable. A solution of 80 mL methylprednisolone was administrated intramuscularly with a recommendation of daily use of betamethasone cream locally on the skin lesion.

**Supplementary Fig. S1:**
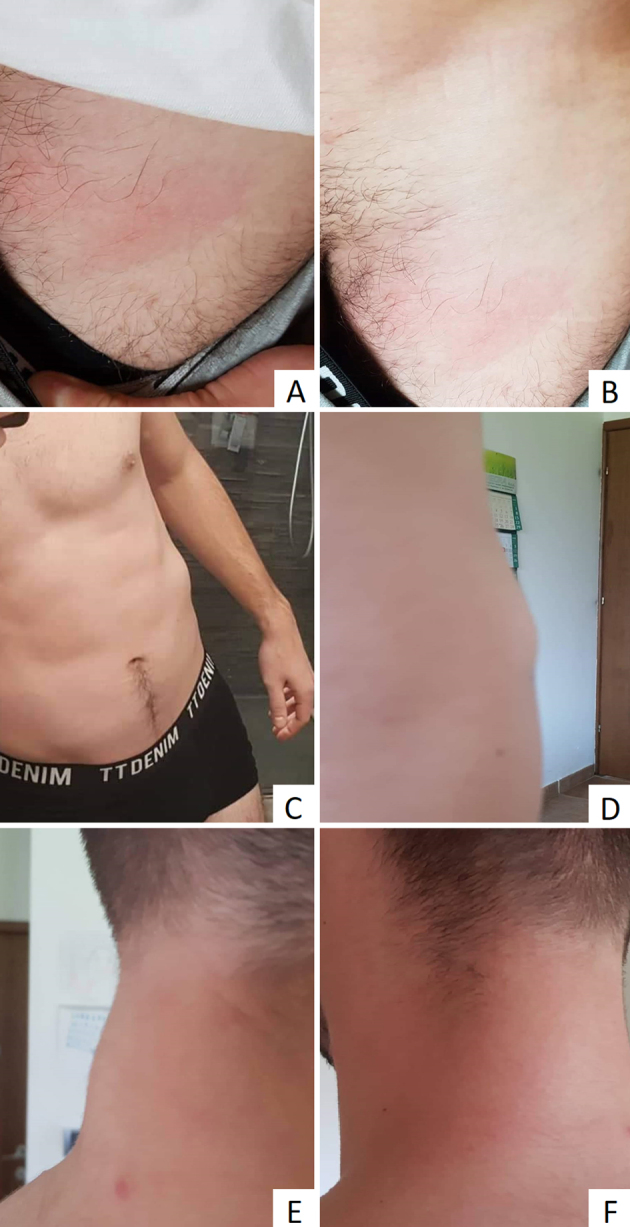
A detailed description of the case and the course of the disease in the patient.

On 7th January 2020, examination of a nodule in the left inguinal region noted a linear plaque measuring 2 × 7 cm (Supplementary Figs. 1A and 1B). The laboratory test showed leukocyte counts of 12.5 cells/μL and 27% eosinophils. All other blood parameters were unremarkable. The patient denied international travel during the previous year but confirmed daily contact with a neighbour's three dogs. Coprological and serologic tests for intestinal and systemic parasitic diseases were subsequently ordered. On 14th January 2020, control examination revealed an increase in leukocyte count of 15.4 cells/μL with 35% eosinophils. All serological assays and coprological tests, repeated three times, were negative.

On 20th January 2020, the plaque in the left inguinal region spontaneously resolved, however, two additional painless, subcutaneous nodules were detected. One oval nodule in the left hypochondrium measured 1 × 2 cm (Supplemental figs. 1C and 1D), and a round nodule in the left axilla measured 1.5 × 1.5 cm. Ultrasonography imaging of the abdominal wall nodule showed a fusiform and elongated hyperechoic structure within the left rectus abdominis muscle. Radiological findings suggested parasitic myositis, so fine needle aspiration (FNA) of the nodule was performed. Eosinophil counts in peripheral blood were raised to 44% corresponding to FNA findings of numerous mature eosinophils, moderate numbers of mature lymphocytes, several macrophages and plasma cells. No parasitic structures were found.

**Fig. 1:**
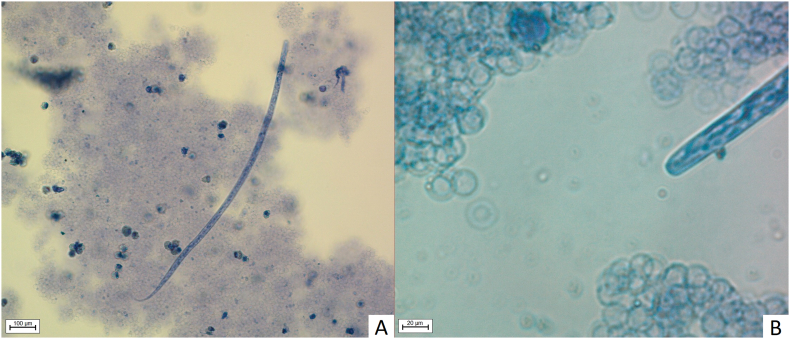
Microfilaria of *D. repens* stained with Diff Quick. 1A: Whole microfilaria; 1B: Anterior part (AxioImager. M2, Zeiss, Jena, Germany).

Blood samples were collected for dirofilariasis screening on 23rd January 2020. Modified Knott's test performed on 6 mL of EDTA blood (6 × 1 mL) revealed the presence of 2 microfilaria/mL that morphologically corresponded to *D. repens* (Fig. 1). The suspected presence of *D. repens* was confirmed using species-specific PCR that amplifies a portion of the cytochrome oxidase subunit 1 (COI) gene [9].

At the same time, skin inspection revealed a new nodule, measuring 1 cm in diameter, located under the intact skin of the neck (Supplementary Figs. 1E and 1F). Surgical excision of all three subcutaneous nodules was performed on 27th January 2020. In preoperative laboratory findings, eosinophilia was evaluated, reaching 48% of white blood cells. A transversal cut surface of two extracted nodules had centrally placed white, slender, filarial worms.

All symptoms resolved within 48 h after surgical removal of parasites. No additional treatment was applied. Over the next three weeks, the patient was regularly monitored for skin lesions, complete and differential blood count and peripheral blood microfilaremia (Table 1). Additionally, blood samples from all three dogs in contact with the patient were collected for dirofilariasis screening.

**Table 1:** Laboratory results of complete leukocyte count, relative lymphocyte and eosinophil count, Knott's test and molecular detection of *Dirofilaria repens* and *Wolbachia* sp. prior and after surgical extraction of nematodes. Date of operative procedure is marked with underline. Wbc – white blood count; Ref – reference; PCR – polymerase chain reaction; Jan – January, Feb – February, Mar – March.

Parameter	Jan 7	Jan 14	Jan 22	Jan 24	Jan 27	Feb 3	Feb 13	Mar 9	May 29
Wbc (x 10^9^/L) Ref range: 4.4–11.6	12.5	15.4	14.8	14.8	13.6	⁎	⁎	⁎	⁎
Lymphocytes (%) Ref range: 19–52	31	22	20.1	18	19	⁎	⁎	⁎	⁎
Eosinophils (%) Ref range: 0–9	27	35	44	48	40	20	28	12	4.5
Knott's test (microfilariae/mL)				12/4.5	0/4	0/6	0/4	0/4	0/4
PCR *D. repens*				+	−	−	−	−	−
PCR *Wolbachia* sp.				+	−	−	−	−	−

⁎ Within reference value.

## 3 Materials and methods

### 3.1 Morphological and molecular examinations

The modified concentration procedure by Knott was performed on EDTA blood samples collected from the human patient and dogs for detection of microfilariae (L1) [1] during the study period.

For species confirmation, parasites or tissue samples were cut in pieces and DNA was extracted using the DNA ‘Blood and tissue kit’ (Qiagen, Hilden, Germany) in the automatic extraction system Qiacube (Qiagen, Hilden, Germany). The same kit was used for extraction of nucleic acids from 200 μL blood samples. Species-specific PCRs that amplify a fragment of approximately 200 bp specific to the COI gene for *D. immitis* (DI COI —F1 AGT GTA GAG GGT CAG CCT GAG TTA and DI COI-R1 ACA GGC ACT GAC AAT ACC AAT) and for *D. repens* (DR COI-F1 AGT GTT GAT GGT CAA CCT GAA TTA and DR COI-R1 GCC AAA ACA GGA ACA GAT AAA ACT) were used in the study [9]. For sequencing, the protocol described by Casiraghi et al. [10] was applied to amplify a 667-bp region of the COI gene. All samples were also screened by conventional PCR for *Anaplasma*/*Ehrlichia* species based on amplification of a 345-bp 16S rRNA gene fragment [11]. The amplified products were analyzed by capillary electrophoresis (QIAxcel System®, QIAGEN) with size markers in the range of 100–2500 bp. Samples were purified with ExoSAP-IT® (USB Corp., Cleveland, United States) and sequenced in both directions by Macrogen Inc. (The Netherlands). Sequences were assembled using the SeqMan Pro software, edited with EditSeq of the Lasergene software (DNASTAR, Madison WI, USA) and compared with available sequences using BLAST.

### 3.2 Literature review

Metadata on cases of human infection with *D. repens* in adult (L5) and microfilarial (L1) stage were analyzed. The studies and their analysis examined in the present work are the result of extensive text mining and searching through electronically available databases (Medline/PubMed, Web Of Science, Embase, Scopus), individual journals and proceedings papers for all results retrieved by searches of any of the keywords: “Zoonosis”, “Vector-borne disease”, “Parasite”, “Helminths”, “Nematode”, “Human”, “Dirofilariasis”, “*Dirofilaria repens*“, “Microfilaria”, “Microfilaremia”, “Microfilariasis”, “Blood”, “Subcutaneous”, “Ocular”, “Eosinophils”, “Eosinophilia”, “Knott”, “PCR”; as well as their combinations. Both cases with *D. repens* microfilariae detected in peripheral blood and/or local tissues were considered. The cross-referenced list of articles included in the review was manually checked for relevant studies. All studies written in English and other than English language were analyzed. After the screening of all identified articles, only those that met the criteria for eligibility were included in the study.

The search retrieved a total number of 19 articles [5,7,8,[12], [13], [14], [15], [16], [17], [18], [19], [20], [21], [22], [23], [24], [25], [26], [27]]. One article was excluded because it was not available [28]. The review contained articles published until May 2021.

## 4 Results

### 4.1 Molecular detection

**Fig. 2:**
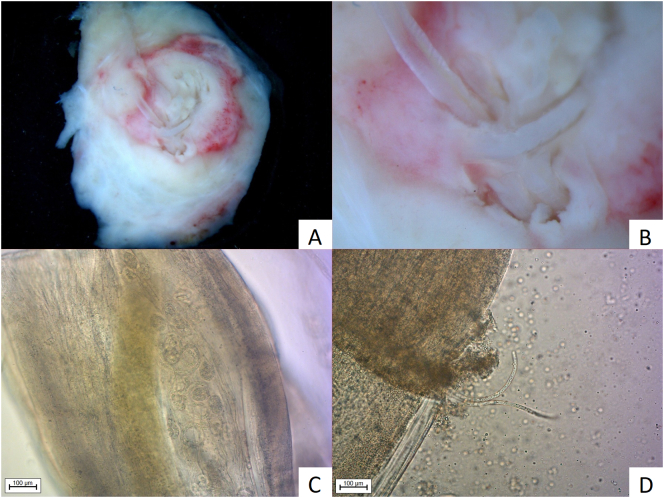
Nodule removed from hypogastric region immersed in physiological saline solution. 2A, 2B: Nodule (5 × 1 cm) with centrally placed *D. repens*; 2C: Microfilariae in uterus of female; 2D: Microfilariae releasing from female (AxioImager. M2, Zeiss, Jena, Germany).

**Fig. 3:**
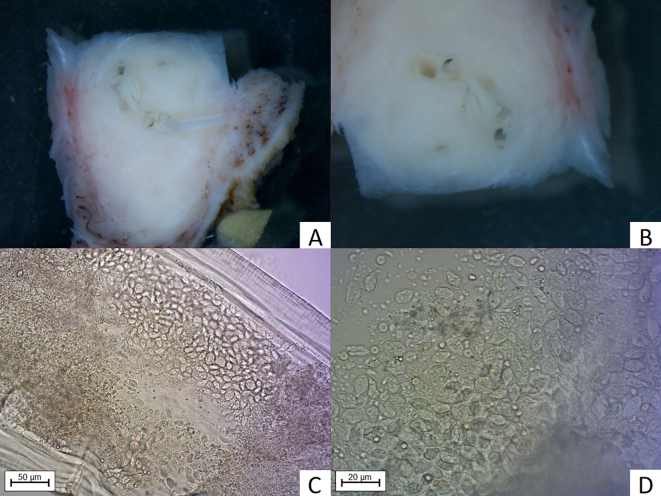
Nodule removed from the axillar region. 3A, 3B: Nodule (2.5 × 1 cm) with centrally placed *D. repens* (StereoDiscovery.V20, Zeiss, Jena, Germany); 3C, 3D: Eggs within uterus of female visible after enlightening with lactophenol (AxioImager. M2, Zeiss, Jena, Germany).

*D. repens* adults were detected in two surgically removed nodules. Microscopic examination revealed the presence of female-producing microfilariae in the nodule from the hypogastic region (Fig. 2), while females isolated from the axillar region were free from microfilariae (Fig. 3). No male parasites were detected. Both amplified sequences (MT847642) were identical to each other but showed two nucleotide difference from Croatian *D. repens* sequences previously detected in a sample obtained from a human scrotum (KX265049). Blood samples were collected from all three pet dogs owned by the patient's neighbour. Microscopic analysis of blood smears after Knott's concentration test revealed 7500 and 8200 microfilariae/mL in two of the dogs, respectively. Identical sequencing results were obtained from both dogs and human patient.

Partial sequences amplified from microfilaria from blood and adult parasites were identical to a *Wolbachia* sp. endosymbiont of *D. repens* previously detected in blood from Croatian dogs [29].

### 4.2 Histopathology

Macroscopically, the tissues removed from the axillar and hypogastric regions were of a soft consistency, measuring 2.5 × 1 cm and 5 × 1 cm and contained centrally placed curled, thin white “structures” 3 mm thick within a narrow canal-like cavity (Fig. 2A and B, Fig. 3A and B). The surrounding panniculus, muscular fibers and dermis showed marked irregular thickening. The samples had a firm consistency with a greyish-red to yellow appearance. The nodular structure removed from the nuchal region on the cut surface revealed a lymph node embedded in edematous subcutaneous tissue.

**Fig. 4:**
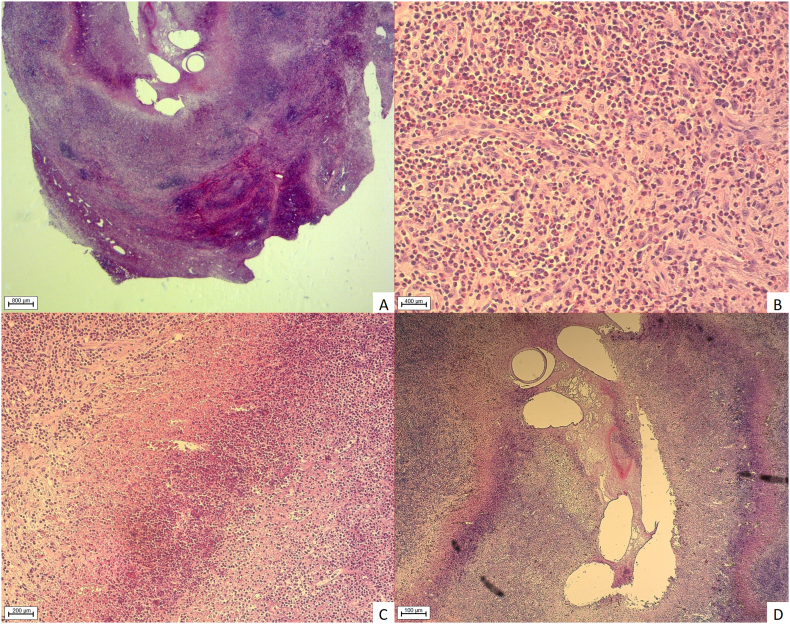
Histopathological examination. 4A: Complete replacement of subcutaneous and muscular tissue by inflammatory cells. 4B: Numerous transversal, tangential and longitudinal sections of blood vessels with pronounced endothelial hyperplasia surrounded by myriad eosinophilic granulocytes. 4C: Border between degenerated inflammatory cells and vital eosinophilic granulocytes. 4D: Transversal cuts through nematode cuticle, artificial empty spaces (loss of nematodes elements) embedded in fibrin, cellular and nuclear debris (AxioImager. M2, Zeiss, Jena, Germany).

Histology of both nodules containing nematodes revealed severe, poorly demarcated infiltration of the subcutaneous and/or skeletal muscle tissue by numerous eosinophilic granulocytes, scattered lymphocytic follicular agglomerations and fewer macrophages, plasma cells, and rare mast cells (Fig. 4A). These infiltrates replaced and extended adipose and/or rectus muscle tissue circumferentially, extending numerous newly formed blood vessels on the lesion periphery (Fig. 4B and C). Many of the newly formed blood vessels showed endothelial hypertrophy embedded within eosinophilic granulocytes cuffs, rare fibroblasts and few lymphoid follicles (Fig. 4C). Massive areas of liquefactive necrosis (Fig. 4D) centrally within both lesions were found harbouring multiple cross and a few longitudinal sections of metazoan parasites, measuring approximately 200–300 μm in diameter. Nematodes showed a 4 μm thick, smooth, eosinophilic cuticle (Fig. 4D). Cuticular ridges were not preserved on longitudinal sections. Coelomyarian musculature was not preserved in the sections, as well as organs of the body cavity.

### 4.3 Literature review

The bibliography search retrieved 19 results between 1992 and 2021, reporting a further 21 human infestations with adult *D. repens* producing microfilariae beyond the case described in this study. A total of 20 (90.91%) cases were from Europe and two (9.09%) from Asian countries. Only four (18.18%) patients had medical record of chronic immune disorder while others were immunocompetent (63.64%) or data were not described (18.18%) (Table 2).

**Table 2:** Epidemiological characteristics of human infection with *D. repens* in adult (L5) and microfilarial (L1) stage (chart review of the world literature). Ref – reference.

Ref	Year	Age	Sex	Country	Travelling history	Immunological status
[17]	1992	53	Female	Italy	Unknown	Malignancy
[18]	1994	50	Male	France	Corsica	Malignancy
[16]	1998	70	Male	Greece	None	Immunocompetent
[12]	2004	60	Male	Russia	None	Immunocompetent
[13]	2005	62	Male	Hungary	None	Malignancy
[19]	2007	40	Male	Iran	None	Immunocompetent
[23]	2009	40	Male	Serbia	None	Immunocompetent
[23]	2009	21	Male	Serbia	None	Immunocompetent
[15]	2009	45	Male	Germany	India; Sri Lanka	Immunocompetent
[22]	2013	63	Female	Russia	Southeast Asia	Immunocompetent
[14]	2013	28	Female	Ukraine	Unknown	Unknown
[14]	2013	65	Female	Ukraine	Unknown	Unknown
[24]	2014	62	Male	India	Unknown	Immunocompetent
[26]	2015	38	Female	France	Tunisia	Immunocompetent
[21]	2016	30	Female	Italy	India; Australia	Immunocompetent
[5]	2016	17–61	Unknown	Czech Republic	Hungary; Slovakia; Croatia	Unknown
[25]	2017	70	Female	France	New Caledonia	Immunocompetent
[8]	2018	28	Male	Poland	None	Immunocompetent
[7]	2018	76	Male	Belgium	Senegal	Diabetes mellitus
[20]	2020	56	Female	Austria	Greece; India	Immunocompetent
[27]	2021	79	Male	Germany	Sri Lanka	Unknown
⁎	2021	17	Male	Croatia	None	Immunocompetent

⁎ current study.

Within the study population, there were 13 (59.09%) males, eight (36.36%) females and, in one (4.55%) case, sex was not reported. The mean age at presentation was 50.14 years, ranging from 17 to 79 years. A total of 11 (50.00%) cases had subcutaneous dirofilariasis, six (27.27%) had ocular dirofiliariasis, with single cases (4.55% each) of genital, mammary, lymphatic and a combination of subcutaneous and pulmonary dirofilariasis described. In one (4.55%) case, the primary anatomical site of adult *D. repens* could not be found. *D. repens* microfilariae were detected in the local tissue (local microfilariasis) in 11 (50.00%) cases and the peripheral blood (microfilaremia) in 11 (50.50%) cases. Samples were obtained by venipuncture in 11 (50.00%) cases, excisional biopsy in eight (36.36%) cases and FNAC in three (13.64%) cases. In cases of microfilaremia, adult *D. repens* were located in subcutaneous tissue in six (54.55%) cases, the eye in four (36.36%) cases and an unknown primary site in one (9.09%) case. The mean value of microfilariae detected on Knott's test was 5.56/mL (range, 1–12/ml). The mean eosinophil count was 2356 cells/μL (range, 1100–6900 cells/μL). Final identification of *D. repens* microfilariae was based on morphological detection (microscopy) in 14 (63.64%) cases, and molecular detection (PCR) in eight (36.36%) cases. Treatment with surgical extirpation of adult *D. repens* was performed in 18 (81.82%) cases and medications were administered in 11 (50.00%) cases, the latter consisting of antiparasitic drugs in nine (40.91%) cases, antibiotics in four (18.18%) cases and corticosteroids in one (4.55%) case. In one (4.55%) case, treatment modality was unknown (Table 3).

**Table 3:** Clinical characteristics of human infection with *D. repens* in adult (L5) and microfilarial (L1) stage (chart review of the world literature). Ref – reference; KT – Knott's test; N – number; FNAC – fine needle aspiration cytology; HPE – histopathological examination; PCR – Polymerase chain reaction.

Ref	*D. repens* L5 location	*D. repens* L1 location	*D. repens* L1 detection	KT (N/ml)	Eosinophils cells/μL	Treatment modality
[17]	Mammary	Local tissue	HPE	/	Eosinophilia	Surgical
[18]	Subcutaneous	Blood	KT	11	Unknown	Unknown
[16]	Unknown	Blood	KT	Several	3000	Diethylcarbamazine
[12]	Subcutaneous	Local tissue	FNAC	/	Unknown	None
[13]	Ocular	Blood	KT	1	Unknown	Surgical; Mebendazol; Levamisolum; Albendazol; Ivermectin
[19]	Subcutaneous	Local tissue	FNAC	/	Eosinophilia	Surgical
[23]	Genital	Local tissue	HPE	/	Unknown	Surgical
[23]	Subcutaneous	Local tissue	HPE	/	Unknown	Surgical
[15]	Subcutaneous	Local tissue	HPE	/	1100	Surgical; Albendazol; Methyl-prednisolone
[22]	Subcutaneous	Blood	KT; PCR	2	1300	Surgical; Doxycycline
[14]	Ocular	Local tissue	HPE	/	Unknown	Surgical
[14]	Ocular	Local tissue	HPE	/	Unknown	Surgical
[24]	Subcutaneous	Local tissue	HPE	/	Unknown	Surgical
[26]	Pulmonary, Subcutaneous	Blood	KT; PCR	1	2300	Surgical; Ivermectin, Albendazole; Diethylcarbamazine
[21]	Subcutaneous	Local tissue	FNAC; PCR	/	1100	Doxycycline; Ivermectin
[5]	Lymphatic	Local tissue	HPE	/	Unknown	Surgical
[25]	Ocular	Blood	KT; PCR	4	Unknown	Surgical; Doxycycline; Ivermectin
[8]	Subcutaneous	Blood	KT, PCR	12	1400	Surgical; Ivermectin; Albendazole
[7]	Ocular	Blood	KT	6	2400	Surgical; Ivermectin
[20]	Subcutaneous	Blood	KT; PCR	4	1700	Surgical; Doxycycline
[27]	Ocular	Blood	PCR	/	Eosinophilia	Surgical; Ivermectin
⁎	Subcutaneous	Blood	KT; PCR	9	6900	Surgical

⁎ Current study.

## 5 Discussion

We report a new case of *D. repens* microfilariemia in the circulatory system of a young and healthy male patient with severe eosinophilia. Clinical manifestation commenced with rash erythema. Differential diagnosis included allergic reaction, cutaneous larvae migrans and malignant neoplasia due to increased leukocyte count and eosinophilia, but with normal IgE titres. Although all tests for parasitic infestation were negative, a constant increase in eosinophils and highly specific findings on ultrasonography of formed subcutaneous nodules raised the suspicion of filarial infection [30]. *D. repens* infection was finally confirmed with morphological and molecular identification of microfilariae from the blood stream [31]. This case also represented, to the best of our knowledge, the first molecular confirmation of *Wolbachia* sp., endosymbionts of *D. repens* from blood, raising questions about the potential use of this approach in the diagnosis of *D. repens* infections.

It is generally considered that human hosts are unsuitable for completion of the *D. repens* life cycle. Based on analysis of 266 human cases, Ermakova et al. concluded that humans are a biological ‘dead-end’ for this helminth [32]. The usual findings involve the detection of a single subadult/adult worm but, on occasion, they may develop to mature adults, mate and produce microfilariae. In rare cases, the microfilariae may even reach the bloodstream [1,3,6]. In the current study, both helminths from skin nodules were morphologically identified as adult females. The worm from the skin nodule in the patient's abdominal wall had developed to maturity and contained microfilariae (Fig. 2) while the female from the axilla did not contain microfilariae (Fig. 3). Detection of a female producing microfilariae indicates the likely presence of a male worm even if it was not specifically detected [1]. In addition to our case, meta-analysis revealed 21 published cases of human infestation with females producing microfilarae since 1992. In half of the cases, microfilariae were detected in the bloodstream with the modified Knott's test while, in others, fertility was confirmed after their detection in worms or tissue. On this basis, and combined with the fact that Ermakova et al. described sexually matured parasites in 10.4% of nodules, the presumption that humans are a ‘dead-end’ for this helminth does not appear to be correct [32]. Although detection of *D. repens* microfilariae in the circulation indicates the likely presence of adult worms of both sexes within the human host, the definitive presence of both adult male and female helminths in human cases remains to be demonstrated. Such evidence will be required to put this question to bed. As to whether this human and other human cases could act as reservoirs of infection, this is less clear. In the current case, the presence of microfilariae in the blood appears to have only been short-lived. Combined with the fact that only a low number of parasites (1–12/mL) could be detected in the blood in this case and in other 10 patients previously described (Table 3), its our assumption that the risk of humans acting as reservoirs of *D. repens* infection is relatively low, certainly at least compared to other hosts such as dogs.

Previously described cases in human patients suggested immunodeficiency as a risk factor for *D. repens* microfilaremia [13,17,18], however, in most cases described in the literature, as well as the current case, the patients were immunocompetent (Table 2). Knowledge of the immunopathogenic mechanisms of dirofilariosis in humans is poorly understood. In the most comprehensive study, eosinophilia appeared in 16.4% patients and was attributed to migration of helminths [32]. In our case, both parasites were settled within necrotic tissue, demarcated but not encapsulated, with massive inflammation and neoangiogenesis of the subcutaneous and/or skeletal muscle tissue. Systemic eosinophilia continued to increase. It is possible that the very high levels of peripheral blood eosinophilia described in this case may have been stimulated by biomediators released from necrotic tissue [33] surrounding the adults delivering microfilaria. The support for this thesis lies in the fact that all previously reported cases with microfilariae developed eosinophilia (Table 3). In terms of the host serological response, in contrast to a study reported by Lechner et al. and in agreement with work by Potters et al., levels of IgE antibodies were within reference ranges in our case [7,20].

Unfortunately, as illustrated in Table 3, the modified Knott's test has been rarely used for detection of microfilariaemia despite their presence within tissue after excisional biopsy or FNA. In order to provide a more accurate insight into the number of patients with circulating microfilariae infected with *D. repens*, the modified Knott's test should be used regularly and in larger blood volumes than 1 mL.

In addition to the surgical removal of both nodules that contained the adult helminths, systemic anti-helminthic or doxycycline therapy was recommended given the presence of microfilariaemia, however, was not actioned by the patient. In order to prevent recurrence of disease, two dogs identified as potential sources of infection were treated and protected with repellent collars [34]. Contrary to previous reports, this is the first microfilaretic human patient treated only surgically without any additional anti-helminthic or antibiotic treatment (Table 3). During follow-up, all blood samples were free from microfilariae 12 months after adults of the *D. repens* extirpation. The extremely high levels of eosinophilia may be potentially responsible for the short duration of microfilariemia. Given the scarce knowledge of a persistence of microfilaria in the human blood stream, the findings from this case suggest transient microfilariaemia and short survival. All previous patients with microfilariemia received medicamentous treatment and it was impossible to otherwise evaluate the duration of microfilariemia.

As already mentioned, the heavily infected dogs with 7500 and 8200 microfilariae/mL are the likely source of infection with continuous exposure to multiple infections probably crucial for development of adult parasites rather than the immune status of patient (Table 2).

## 6 Conclusion

To our knowledge, this is a first report on the molecular identification of multiple *D. repens* adults, *Wolbachia* sp. and microfilariae in a human patient, with suspected transmission from infected dogs. Despite the fact that dirofilariasis is one of the most important parasitic diseases emerging in humans and dogs across Europe, it is still neglected and rarely considered as cause of human ocular and dermatological manifestations as described in the current case. The evidence of the current case provides evidence to support a role for humans as definitive hosts of *D. repens*. From a diagnostic perspective, regular use of modified Knott's test in humans and dogs should be adopted when investigating human clinical presentations with symptoms that may be consistent with filariasis [35]. More broadly, this study highlights the crucial value of a One Health approach to further dissecting the host range and transmission of this neglected vector-borne filaroid helminth.

Supplementary data to this article can be found online at [doi].

Supplementary material Image 1

## Ethics approval and patient consent

The authors assert that all procedures contributing to this work were approved and complied with the ethical standards of the relevant national and institutional committees on human experimentation and with the Declaration of Helsinki 1964 (revised in 2013).

Written consent was provided by the patient and his parents to enable anonymized reporting of the results of this study.

## Availability of data and materials

The data generated during this study are included within this manuscript or are available upon request from the corresponding author.

## Financial support

This research did not receive any specific grant from funding agencies in the public, commercial, or not-for-profit sectors.

## Authors' contributions

Conceptualization, R.B. and A.P·B.; methodology, R.B., A.B. and J.P.B.; software, D.J.; validation, R.B., A.B. and A.P.; formal analysis, R.B. and D.J.; investigation, A.P.B. and A.B.; resources, R.B.; data curation, J.P.B.; writing—original draft preparation, A.P.B., J.P.B, R.B. and A.B.; writing—review and editing, A.P.; visualization, J.P.B.; supervision, A.P. All authors have read and agreed to the published version of the manuscript.

## Declaration of Competing Interest

None.
