# Supplementary material for: Dirofilaria repens microfilaremia in humans: Case description and literature review
Source: One Health. 2021 Aug 12;13:100306. doi: 10.1016/j.onehlt.2021.100306 (PMC8385151; doi:10.1016/j.onehlt.2021.100306)
Supplement: Supplementary file 1 — Supplementary material [file mmc2.docx]

**CASE DESCRIPTION**

A 17-year old adolescent athlete presented at an emergency room on the 10^th^ December 2019 due to an acute onset of burning pain in the left inguinal region lasting for two hours, followed by formation of a shallow nodule under the intact skin. The nodule was warm, red, painless on pressure and not pruritic, measuring 5 x 3 cm in size. The patient was subfebrile (37.2 °C), in a good general condition without any previous medical history, reporting only diarrhoea in the days prior to the onset of dermatological issues. Regional lymph nodes and blood test findings were unremarkable, herniation was excluded and diagnosis of cellulitis of unknown aetiology was made. A solution of 80 mL methylprednisolone was administrated intramuscularly with a recommendation of daily use of betamethasone cream locally on the skin lesion. On the 20^th^ December 2019, after 10 days of local betamethasone cream treatment, a dermatologist recorded intermittent urtica-like lesions appearing all over the left side of the thoracic skin. Betamethasone cream treatment reduced erythema on new lesions in 2 – 3 days but disseminated oval plaques less than 1 cm in diameter remained. The patient denied allergies to food and drugs with a skin prick test confirming negative results. Blood tests showed elevation of eosinophil to 16%, but all other parameters were unremarkable, including the total level of IgE (25.77 IU/mL). Due to a diagnosis “other prurigo” (ICD-10-CM Code: L28.2), betamethasone cream was administered locally on every new urtica-like lesion and expanded to include peroral treatment with 5 mg desloratadine twice a day. The finding of mild eosinophilia prompted the recommendation for stool screening for parasitic infection.

On the 7th January 2020, examination of a nodule in the left inguinal region changed to a linear plaque measuring 2 x 7cm (Supplemental figures 1A and 1B). The plaque was painless, erythematous, mildly pruritic and still accompanied by several irregular multifocal intermittent urtica skin lesions on the left thorax. The patient denied travel outside of Croatia during the previous year, but confirmed daily contact with three neighbour’s dogs in his residence in Zagreb County, Croatia. The laboratory test showed leukocyte counts of 12.5 cells/μL and 27% eosinophils. All other blood parameters were unremarkable. Intestinal parasitosis with cutaneous larva migrans or systemic parasitosis was suspected and stool and serologic tests for intestinal and systemic parasitic diseases were ordered (trichinosis, toxocariasis, schistosomiasis, distomatosis, cysticercosis, toxoplasmosis, echinococcosis, entamoebasis, ascariosis). A preliminary diagnosis of exanthema and eosinophilia due to parasitosis was made. Symptomatic therapy to reduce cutaneous erythema continued.

On the 14th January 2020, control examination revealed an increase in leukocyte count of 15.4 cells/μL with 35% eosinophils. All serological assays and coprological tests, repeated three times, were negative. Thoracic radiography and abdominal ultrasound findings were unremarkable. Diagnosis of eosinophilia of unknown aetiology was established.

On the 20th January 2020, the plaque in the left inguinal region spontaneously resolved, leaving few discrete subcutaneous nodules measuring a few millimetres in diameter. Intermittent urticate present on the thoracic skin resolved as well, but two additional painless, subcutaneous nodules were detected. One oval nodule in the left hypochondrium measured 1 x 2 cm (Supplemental figures 1C and 1D), and a round nodule in the left axilla measured 1.5 x 1.5 cm. Ultrasonography imaging of the abdominal wall nodule showed a fusiform and elongated hyperechoic structure within the left rectus abdominis muscle. The structure had parallel walls exhibiting continuous oscillating to-and-fro movement. Radiological findings suggested parasitic myositis, most likely filariasis, so fine needle aspiration (FNA) of the nodule was recommended.

On the 22^th^ January 2020, using local anaesthesia and guided by the ultrasound, FNA of the nodule in the left rectus abdominis muscle was performed. Eosinophils count in peripheral blood raised to 44% corresponding to FNA findings of numerous mature eosinophils, numerous free eosinophilic granules admixed with moderate numbers of mature lymphocytes, several macrophages and plasma cells. No parasitic structures were found. The background of the cytological smear contained large amounts of granular cellular debris. Findings suggested eosinophilic and lymphocytic inflammation within the subcutaneous nodule. A diagnosis of eosinophilia of unknown aetiology remained with suggestions to conduct further searches for a potential infectious agent.

Blood samples were collected and sent to the Croatian Veterinary Institute for dirofilariasis screening on the 23^rd^ January 2020. Light microscopic examination of 6 mL of concentrated blood samples using Knott's method detected circulating microfilariae corresponding to *D. repens* after morphometry and genotyping (Figure 1).

At the same time, the infectious disease specialist repeated all serologic tests previously performed for intestinal and systemic parasitic diseases with all results negative again. Skin inspection revealed a new third nodule, measuring 1 cm in diameter, located under the intact skin of the nuchal region on the left side of the neck (Supplemental figures 1E and 1F). This nodule was elastic and painful on palpation. The previously described nodules, one on the left hypochondrium and the other in the axillar region, were of rubbery consistency and unpainful. Since FNA failed to detect parasitic elements within the inspected node and all repeated serologic and coprology tests were negative, a diagnosis of lymphoproliferative disease was included in the differential diagnosis.

On the 24^th^ January 2020, after case review by a haematologist-oncologist specialist, surgical excision of all nodules was requested. Modified Knott’s test performed on 6 mL of EDTA blood (6 x 1 ml) revealed the presence of 2 microfilaria/mL that morphologically corresponded to ones of *D. repens*. The suspected presence of *D. repens* was confirmed using species-specific PCR that amplifies a portion of the cytochrome oxidase subunit 1 (COI) gene (Rishniw et al. 2006) [9].

Surgical excision of nodules was performed on the 27^th^ January 2020. In preoperative laboratory findings, eosinophilia was evaluated, reaching 48% of white blood cells. Each nodule was dissected and cut in half. A transversal cut surface of two of the three extracted nodules had centrally placed white, slender, filarial worms surrounded by oedematous subcutaneous tissue. One half of the nodules were placed in formalin for routine histopathology to exclude neoplasm and the second half sent in physiological solution to the Croatian Veterinary Institute, Zagreb for further morphological and genetic examinations of the filarid worms and additional histological analyses of host – parasite interaction in order to explain the presence of microfilariemia. After surgery, peripheral blood samples were collected in multiple time intervals during the morning for examination of the presence of microfilariae.

All symptoms resolved within 48 hours after surgical removal of parasites. No additional treatment was applied. Over the next three weeks, the patient was regularly monitored for skin lesions, complete and differential blood count and peripheral blood microfilaremia (Table 1). No complications or recurrences were observed over the following 12 months with eosinophils returning to their normal range by the 29^th^ May 2020. In addition to detecting the source of infection in this described human case, blood samples from all three dogs in contact with the patient were collected for dirofilariasis screening.
